# Sirt3-mediated mitophagy protects tumor cells against apoptosis under hypoxia

**DOI:** 10.18632/oncotarget.9717

**Published:** 2016-05-30

**Authors:** Aimin Qiao, Kuansong Wang, Yunsheng Yuan, Yidi Guan, Xingcong Ren, Lanya Li, Xisha Chen, Feng Li, Alex F. Chen, Jianda Zhou, Jin-Ming Yang, Yan Cheng

**Affiliations:** ^1^ Center for Bioresources and Drug Discovery and School of Bioscience and Biopharmaceutics, Guangdong Pharmaceutical University, Guangzhou 510006, China; ^2^ Department of Pharmacology, School of Pharmaceutical Sciences, Central South University, Changsha 410008, China; ^3^ Department of Pharmacology, The Penn State Hershey Cancer Institute, The Pennsylvania State University College of Medicine and Milton S. Hershey Medical Center, Hershey, PA 17033, USA; ^4^ Department of Pathology, Xiangya Hospital, Central South University, Changsha 410008, China; ^5^ Department of Pathology, Basic Medical School, Central South University, Changsha 410008, China; ^6^ Department of Plastic Surgery, The Third Xiangya Hospital, Central South University, Changsha 410008, China; ^7^ Engineering Research Center of Cell and Therapeutic Antibody, Ministry of Education, School of Pharmacy, Shanghai Jiao Tong University, Shanghai 200240, China; ^8^ Center for Vascular and Translational Medicine, The College of Pharmacy, Central South University, Changsha 410013, China; ^9^ The Third Xiangya Hospital, Central South University, Changsha 410013, China

**Keywords:** Sirt3, mitophagy, hypoxia, apoptosis, cancer cells

## Abstract

Sirt3, a mitochondrial deacetylase, participates in the regulation of multiple cellular processes through its effect on protein acetylation. The objective of this study was to explore the role of Sirt3 in the mitochondrial autophagy (mitophagy), a process of the specific autophagic elimination of damaged mitochondria. We found that silencing of Sirt3 expression in human glioma cells by RNA interference blunted the hypoxia-induced the localization of LC3 on the mitochondria, and the degradation of mitochondria. These results suggest an important involvement of this protein deacetylase in the induction of mitophagy in cancer cells subjected to hypoxia. Further, we demonstrated that Sirt3 activated the hypoxia-induced mitophagy by increasing the interaction of VDAC1 with Parkin. In the cells subjected to hypoxia, inhibition of Sirt3-mediated mitophagy further decreased the mitochondrial membrane potential, and increased the accumulation of ROS that triggers the degradation of anti-apoptotic proteins Mcl-1 and survivin through the proteasomal pathway. Silencing of Sirt3 expression also promoted apoptosis, and enhanced the sensitivity of cancer cells to hypoxia. The regulatory role of Sirt3 in autophagy and apoptosis was also observed in human breast cancer cells. The results of the current study reveal Sirt3 as a novel regulator coupling mitophagy and apoptosis, two important cellular processes that determine cellular survival and death.

## INTRODUCTION

Sirt3, a member of the sirtuins family of nicotinamide adenine dinucleotide (NAD^+^)-dependent protein deacetylases, mainly localizes at the mitochondria, and controls the acetylation levels of the mitochondrial proteins [1]. Sirt3 has been shown to be implicated in the regulation of a variety of cellular processes, including aging, energy metabolism and stress response, through its effect on protein acetylation [2–5]. For example, Sirt3 is required for the prevention of age-related hearing loss, and the reduction of oxidative stress and damage mediated by the caloric restriction [4, 5]. Sirt3 can protect cells against various stress-induced cell deaths by maintaining the mitochondrial integrity [6, 7]. It was reported that inhibition of Sirt3 increased hypoxia and staurosporine-induced cell death by activating mitochondrial apoptotic pathway [6]. In addition, suppression of Sirt3 expression enhanced the sensitivity of mitochondria to induction of mitochondrial permeability transition (MPT), and potentiated TNF-induced cytotoxicity in cells exposed to ethanol [7].

Autophagy is an evolutionary conserved process in which cellular components are engulfed into a double-membrane autophagosome, and subsequently delivered to the lysosome for degradation. Autophagy occurs constitutively in various types of cells, and contributes to cellular homeostasis through maintaining quality control of both proteins and organelles. Autophagy can be induced in response to various stresses such as starvation, hypoxia and cytotoxic insult [8, 9]. Mitophagy, the specific autophagic elimination of mitochondria, regulates mitochondrion number and maintains quality control of this important organelle [10]. Mitophagy has been shown to contribute to cellular survival by targeting the removal of the damaged mitochondria, eliminating the source of apoptogenic signals or reducing ROS levels. Recently, several pathways involved in mitophagy regulation have been identified in mammalian cells, including BNIP3L, ULK1, ATG7, PINK1 and Parkin [11]. *PINK1* and *parkin* genes are mutated in autosomal recessive Parkinson's disease, thus the defects in mitophagy is believed to be linked to Parkinson's disease. In this study, we intended to determine the roles of Sirt3 in regulating autophagy and apoptosis in tumor cells undergoing stress. We showed that Sirt3 is important for the clearance of the damaged mitochondria through activating mitophagy, and silencing of Sirt3 expression can enhance the sensitivity of tumor cells to stress by inhibiting autophagy and promoting apoptosis.

## RESULTS

### Sirt3 is a positive regulator of autophagy, and inhibition of Sirt3 down-regulates autophagy induced by hypoxia in glioma cells

**Figure 1:**
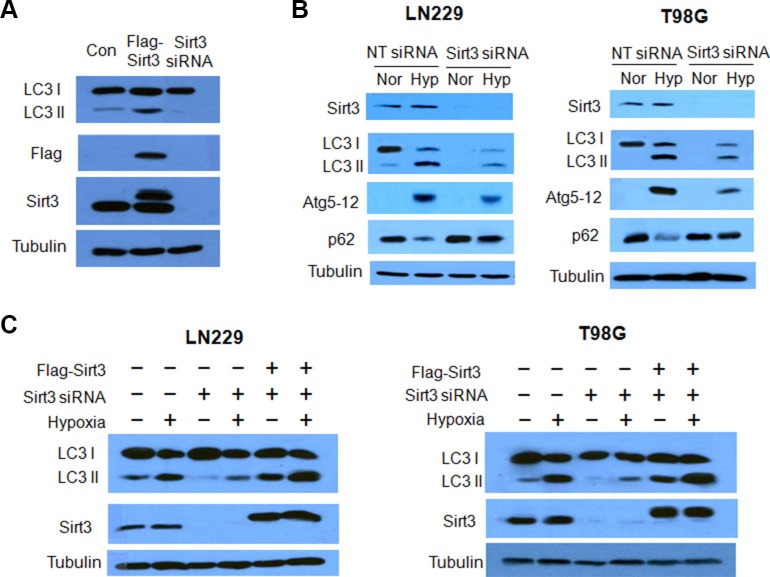
Effects of Sirt3 on hypoxia-induced autophagy in tumor cells **(A)** The human glioma cells LN229 were transfected with a Flag-Sirt3 expression plasmid or a Sirt3 siRNA. The levels of LC3, Flag and Sirt3 were examined by western blot. Tubulin was used as a loading control. **(B)** LN229 and T98G cells were transfected with a non-targeting RNA or a siRNA targeting Sirt3, followed by hypoxia treatment for 24 h. The levels of Sirt3, LC3, Atg5-12 and p62 were examined by Western blot. Tubulin was used as a loading control. **(C)** LN229 or T98G cells were transfected with a Sirt3 siRNA that targets only the noncoding sequences of the Sirt3 mRNA, followed by transfection with a Flag-Sirt3 expression plasmid. The protein levels of LC3 and Sirt3 were examined by western blot. Tubulin was used as a loading control.

To examine the effect of Sirt3 on autophagy, we first compared the amount of LC3 protein in the cells with overexpression of Sirt3 or with silencing of Sirt3 expression. Figure 1A shows that LC3-II protein level was increased when Sirt3 was overexpressed, as compared with that in the control cells; LC3-II protein level was decreased in the cells transfected with a Sirt3 siRNA. We further found that Sirt3 was involved in the activation of autophagy induced by hypoxia in human glioma cells. Silencing of Sirt3 expression markedly blunted autophagic response in the tumor cells subjected to hypoxia, as determined by a decrease in LC3-II and Atg5–12 complex, and an increase in p62 protein (Figure 1B). To validate the effect of Sirt3 on induction of autophagy, we re-introduced Sirt3 into Sirt3 knockdown cells by transfecting a Sirt3 expression plasmid, and then measured autophagic activity following exposure to hypoxia. As shown in Figure 1C, the amount of LC3-II protein was decreased in the hypoxic cells when Sirt3 expression was knocked down; however, introduction of the Sirt3 expression plasmid blocked the down-regulation of LC3-II protein in the cells subjected to silencing of Sirt3 expression. These results indicate that Sirt3 acts as a positive regulator of autophagy in the hypoxia tumor cells.

### Sirt3 activates mitophagy in hypoxic tumor cells

**Figure 2:**
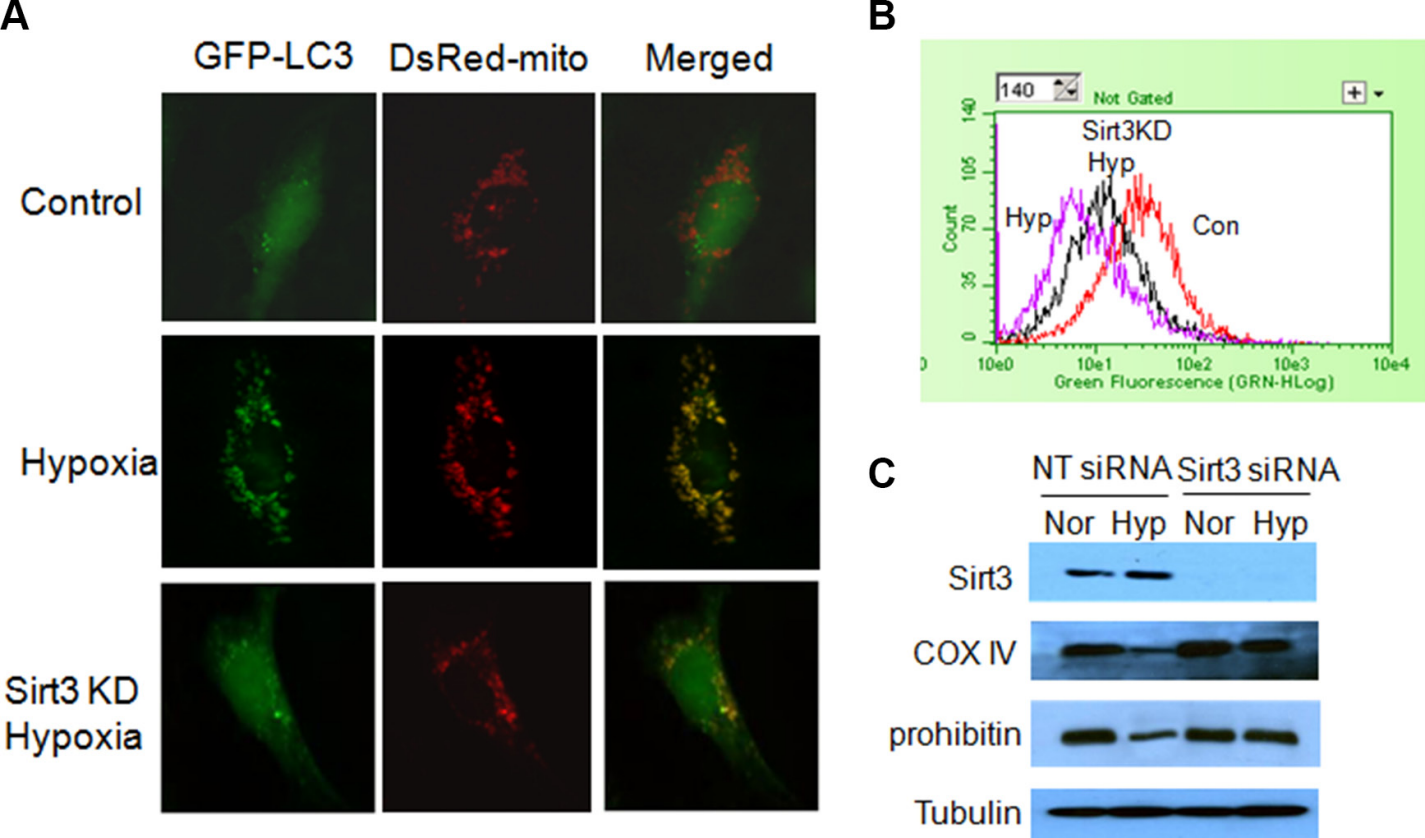
Effect of Sirt3 on mitophagy induced by hypoxia (**A**) LN229 cells with or without Sirt3 silencing were co-transfected with GFP-LC3 and DsRed-mito plasmids, and then exposed to hypoxia for 24 h. The cell morphology was observed under a fluorescence microscopy. LN229 cells were transfected with a non-targeting RNA or a siRNA targeting Sirt3, followed by hypoxia treatment for 24 h. (**B**) The mitochondrial mass was measured by staining with NAO and analyzing by flow cytometry using an Easy Cyteä Plus System (Millipore). (**C**) The levels of Sirt3, COX IV and prohibitin were examined by Western blot. Tubulin was used as a loading control.

**Figure 3:**
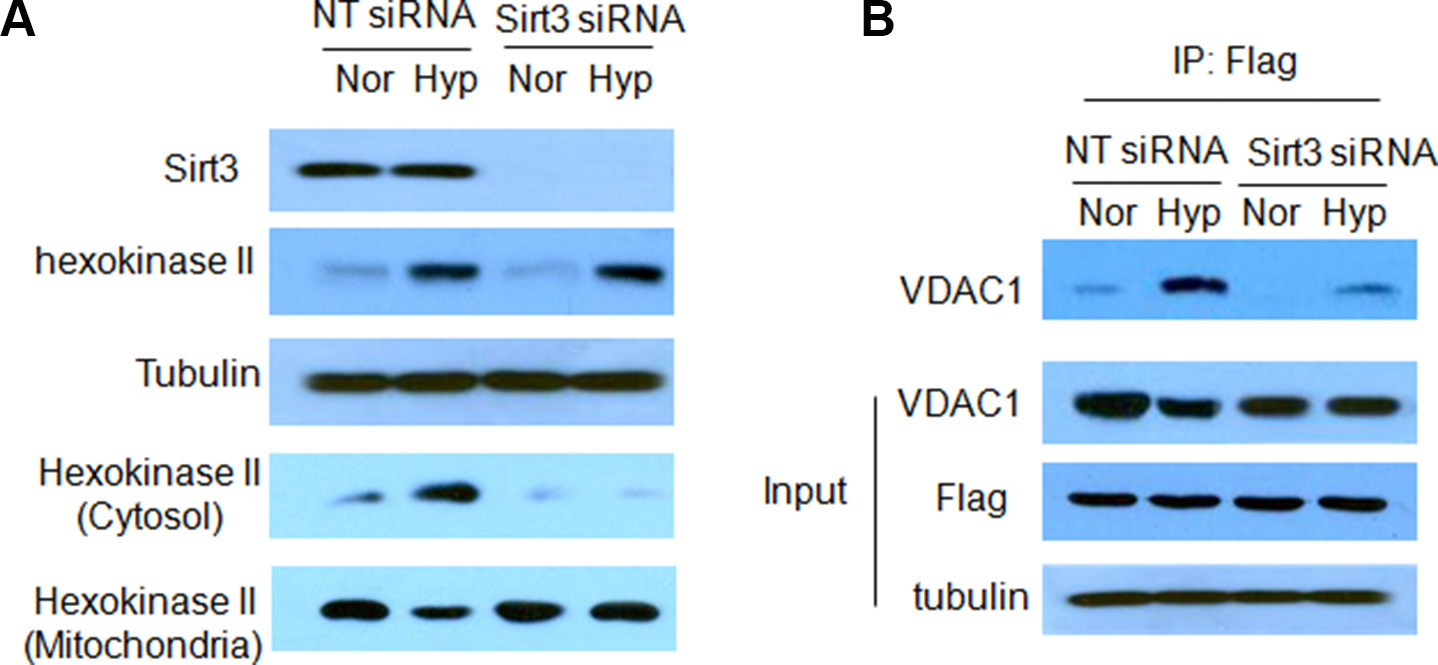
Sirt3 activates mitophagy by enhancing the association of Parkin with VDAC1 (**A**) LN229 cells were transfected with a non-targeting RNA or a siRNA targeting Sirt3, followed by hypoxia treatment for 24 h. The levels of Sirt3 and hexokinase II were examined by Western blot. Tubulin was used as a loading control. The protein levels of hexokinase II in cytosol and mitochondrial fractions were examined by western blot. (**B**) Lysates of LN229 cells transfected with a Flag-tagged Parkin plasmid were immunoprecipitated with a Flag antibody, and the immunoprecipitates were then subjected to western blotting with an anti-VDAC1 antibody.

We recently reported that loss of Sirt3 deteriorated the mtDNA damage and mitochondrial dysfunction caused by irradiation [12]. As mitophagy is activated to degrade damaged mitochondria, we wanted to know whether Sirt3 has a role in the induction of mitophagy. We found that there was a co-localization of GFP-LC3 puncta with mitochondria (red) in tumor cells subjected to hypoxia (Figure 2A), and inhibition of Sirt3 decreased the localization of LC3 on mitochondria (Figure 2A). We then measured the mitochondrial mass by staining cells with nonylacridine orange (NAO), a metachromatic dye that binds to cardiolopin in the mitochondria regardless of their energetic state or membrane potential [13]. As shown in Figure 2B, hypoxia led to a decrease in the mitochondrial mass, and inhibition of Sirt3 blunted the reduction of mitochondrial mass induced by hypoxia. In agreement with these observations, suppression of Sirt3 also blocked hypoxia-induced loss of mitochondrial proteins such as COX IV and prohibitin (Figure 2C). These results suggest that Sirt3 is involved in the selective loss of mitochondria by the hypoxia-induced autophagy. We next wanted to know how Sirt3 activates mitophagy. It has been reported that Sirt3 induces detachment of hexokinase II with VDAC1, a protein localizes to the outer surface of mitochondria [14]. VDAC1 is the mitochondrial target of Parkin, and is essential for Parkin-mediated mitophagy [15]. Here, we found that hypoxia increased the level of hexokinase II expression, and Sirt3 had no effect on the expression of hexokinase II protein (Figure 3A). Interestingly, inhibition of Sirt3 blocked the redistribution of hexokinase II from the mitochondria to the cytosol induced by hypoxia (Figure 3A). Furthermore, the amplified binding of VDAC1 with Parkin in the hypoxic cells was inhibited in the cells with silencing of Sirt3 expression (Figure 3B). These results indicate that Sirt3 may activate mitophagy through decreasing the interaction of VDAC1 with hexokinase II but increasing the interaction of VDAC1 with Parkin.

### Suppression of Sirt3 augments mitochondrial damage, and promotes apoptosis in glioma cells subjected to hypoxia

**Figure 4:**
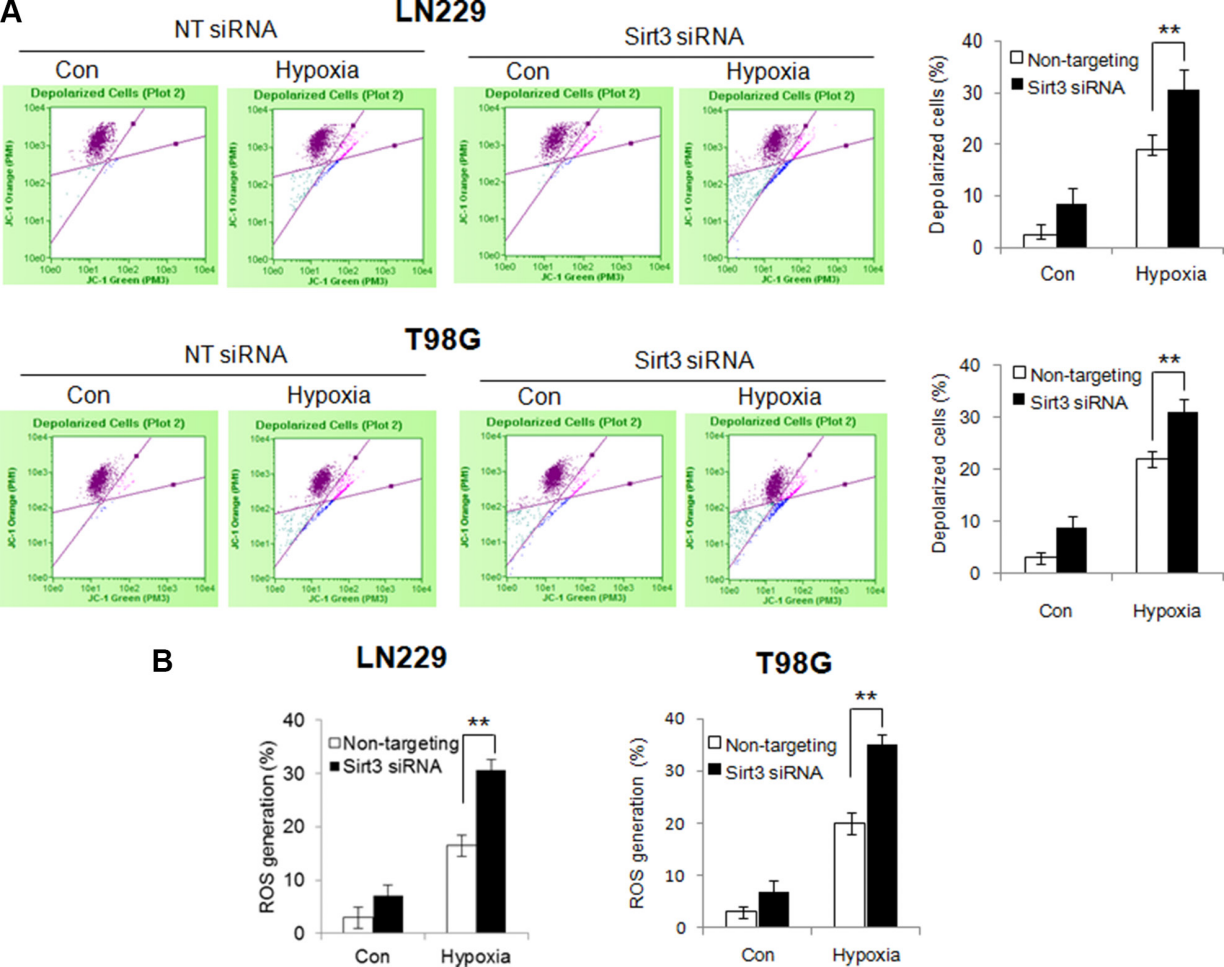
Silencing of Sirt3 expression promotes the reduction of mitochondrial membrane potential and release of ROS in hypoxic glioma cells (**A**) LN229 and T98G cells were transfected with a non-targeting RNA or a siRNA targeting Sirt3, followed by hypoxia treatment. Mitochondrial membrane potential was measured by JC-1 staining using flow cytometry. (**B**) The level of ROS was measured by staining with DCF-DA and flow cytometric analysis.

**Figure 5:**
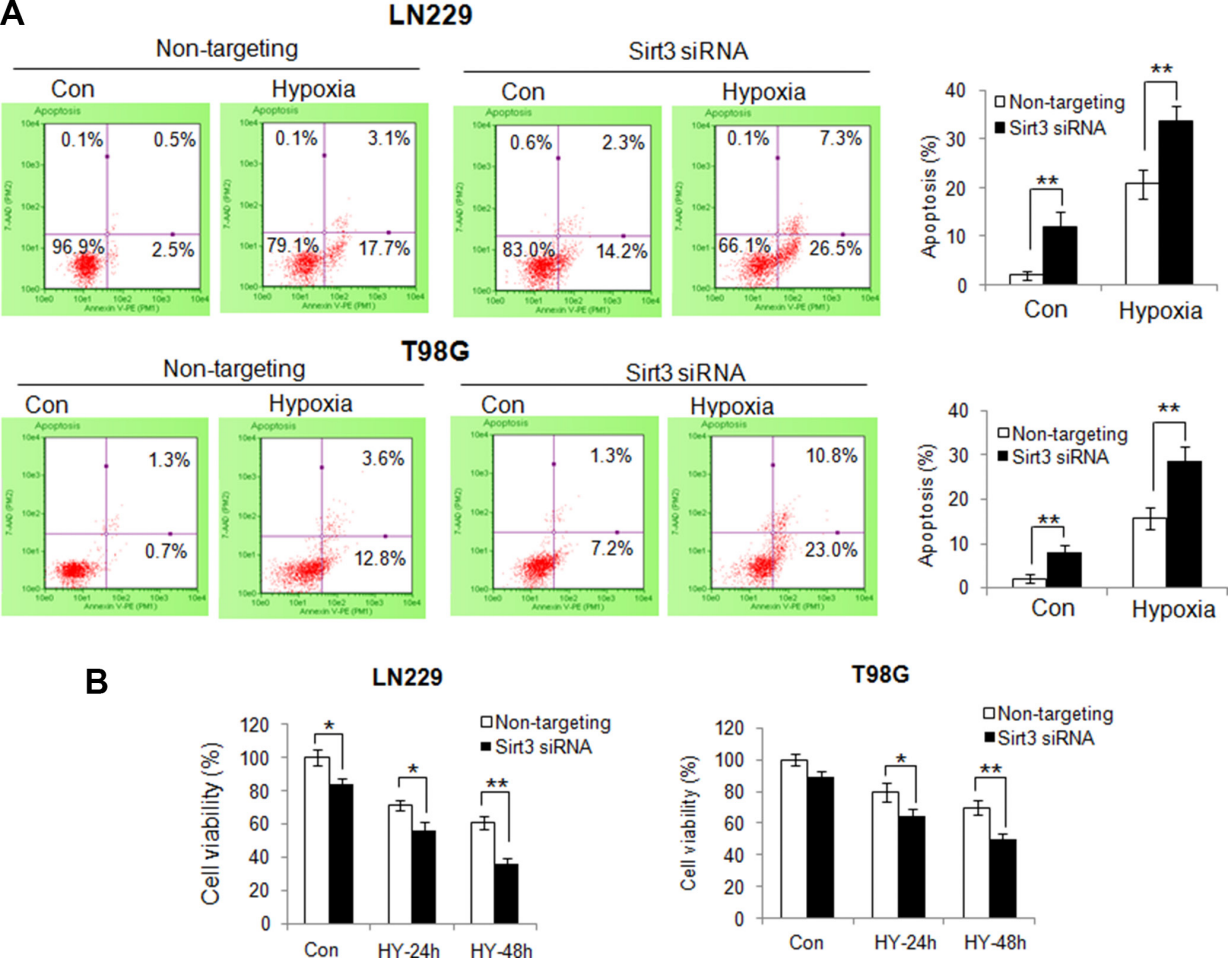
Suppression of Sirt3 augments the activation of apoptosis and increases the sensitivity of tumor cells to hypoxia (**A**) LN229 and T98G cells were transfected with a non-targeting RNA or a siRNA targeting Sirt3, followed by hypoxia treatment for 48h. Apoptosis was determined by flow cytometric analyses of Annexin staining. (**B**) LN229 and T98G cells were transfected with a non-targeting siRNA or a Sirt3-targeted siRNA, followed by hypoxia treatment for 24 or 48h. At the end of treatment, cell viability was measured by MTT assay. Results shown were mean ± SD of quadruplicate determinations from one of three identical experiments.

To determine the consequence of inhibiting mitophagy caused by depletion of Sirt3, we evaluate the mitochondrial function in the cells with silencing of Sirt3 expression. Compared with that in the control cells, hypoxia caused a greater reduction of the mitochondrial membrane potential in the Sirt3-knockdown cells (Figure 4A). In addition, ROS levels were significantly higher in the Sirt3-knockdown cells than in the control cells (Figure 4B). These results indicate that inhibition of Sirt3-mediated mitophagy causes greater mitochondrial damage and more release of ROS from mitochondria. Because mitochondrial dysfunction may trigger apoptosis, we next measured and compared apoptotic activity in the cells with or without depletion of Sirt3 following hypoxia treatment. As shown in Figure 5A, depletion of Sirt3 augmented apoptosis in the tumor cells treated with hypoxia, as evidenced by an increase in Annexin V staining. Furthermore, we found that suppression of Sirt3 substantially increased the sensitivity of glioma cells to hypoxia (Figure 5B). Weighted linear regression analysis informed that the increased tumor cell sensitivity to hypoxia caused by Sirt3 knockdown is significant (*p* < 0.05). These results indicate that inhibition of Sirt3 worsens the mitochondrial damage and activates apoptosis in cancer cells subjected to hypoxia.

### Silencing of Sirt3 expression promotes the proteasomal degradation of Mcl-1 and survivin in a ROS-dependent manner

**Figure 6:**
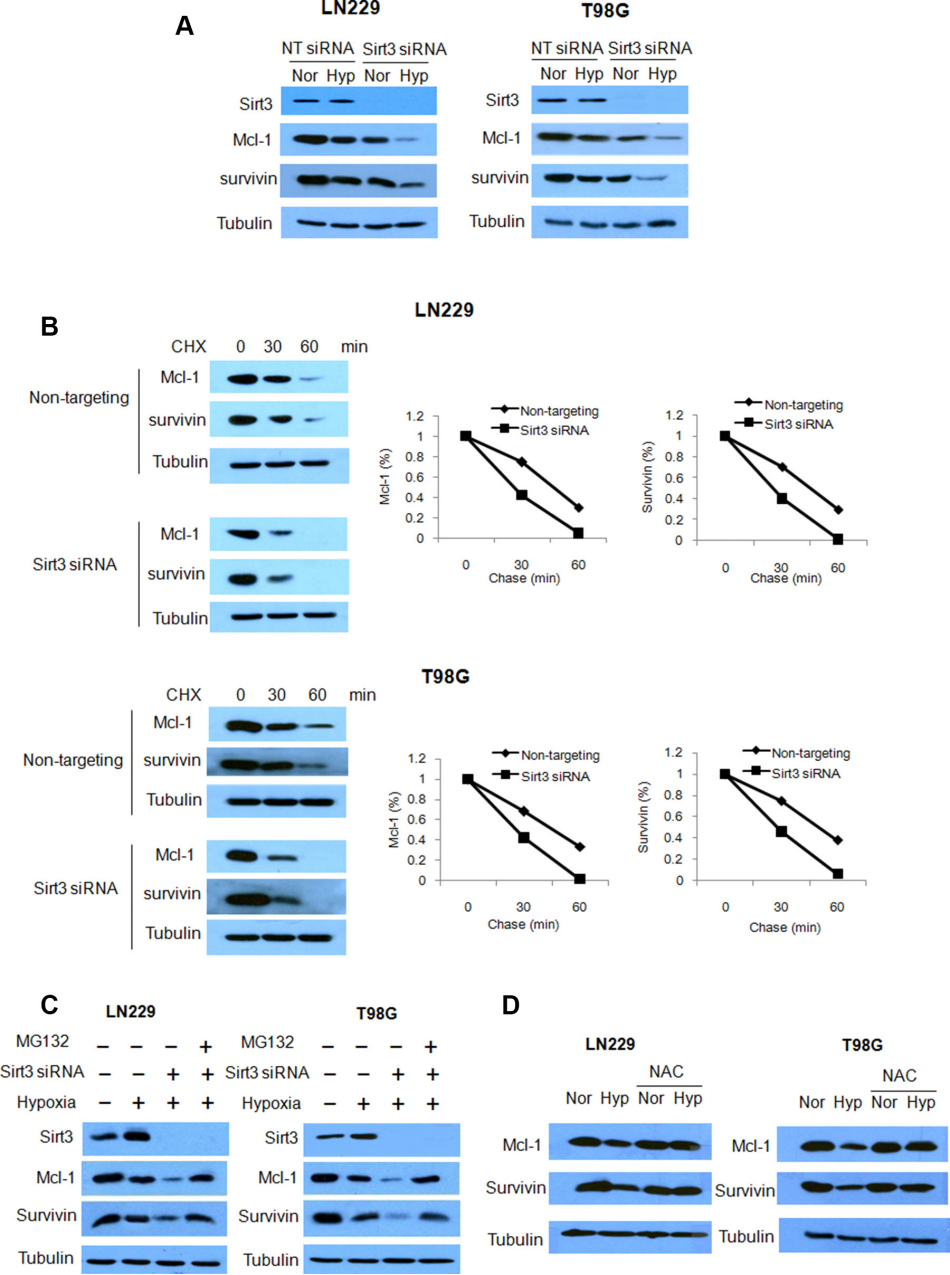
Inhibition of Sirt3 promotes the degradation of Mcl-1 and survivin (**A**) LN229 and T98G cells were transfected with a non-targeting or a Sirt3 siRNA. The expressions of Sirt3, Mcl-1 and survivin were examined by Western blot. Tubulin was used as a loading control. (**B**) LN229 and T98G cells were transfected with a non-targeting or a Sirt3 siRNA, followed by CHX treatment. Mcl-1 and survivin proteins expressions were examined by Western blot. Tubulin was used as a loading control. (**C**) LN229 and T98G cells were transfected with a non-targeting or a Sirt3 siRNA, followed by hypoxia treatment in the presence of MG132. Sirt3, Mcl-1 and survivin protein expressions were examined by Western blot. Tubulin was used as a loading control. (**D**) LN229 and T98G cells were treated with hypoxia in the presence or absence of NAC. Mcl-1 and survivin protein levels were examined by Western blot. Tubulin was used as a loading control.

To explore how depletion of Sirt3 activates apoptosis, we analyzed the expression levels of various apoptosis-related proteins. We observed that the protein levels of Mcl-1 and survivin were further decreased when Sirt3 expression was knocked down in glioma cells subjected to hypoxia (Figure 6A). To determine the mechanism responsible for the changes in amounts of Mcl-1 and survivin proteins, we tested the effects of knockdown of Sirt3 on the expression and degradation of Mcl-1 and survivin proteins. We measured the protein half-lives by cycloheximide chase assays, and found that Sirt3 knockdown dramatically reduced Mcl-1 and survivin half-lives (Figure 6B). These results indicate that Sirt3 has a role in stabilizing of Mcl-1 and survivin proteins. We further demonstrated that proteasome inhibitor, MG132, could rescue the down-regulation of Mcl-1 and survivin in the cells transfected with a Sirt3 siRNA (Figure 6C). In addition, we found that pretreatment with the ROS inhibitor, NAC, blocked hypoxia-induced down-regulations of Mcl-1 and survivin proteins (Figure 6D). These observation suggest that inhibition of Sirt3 may promote the degradation of Mcl-1 and survivin via a ROS-dependent proteasomal pathway.

### Effects of Sirt3 on autophagy and apoptosis in human breast cancer cells

**Figure 7:**
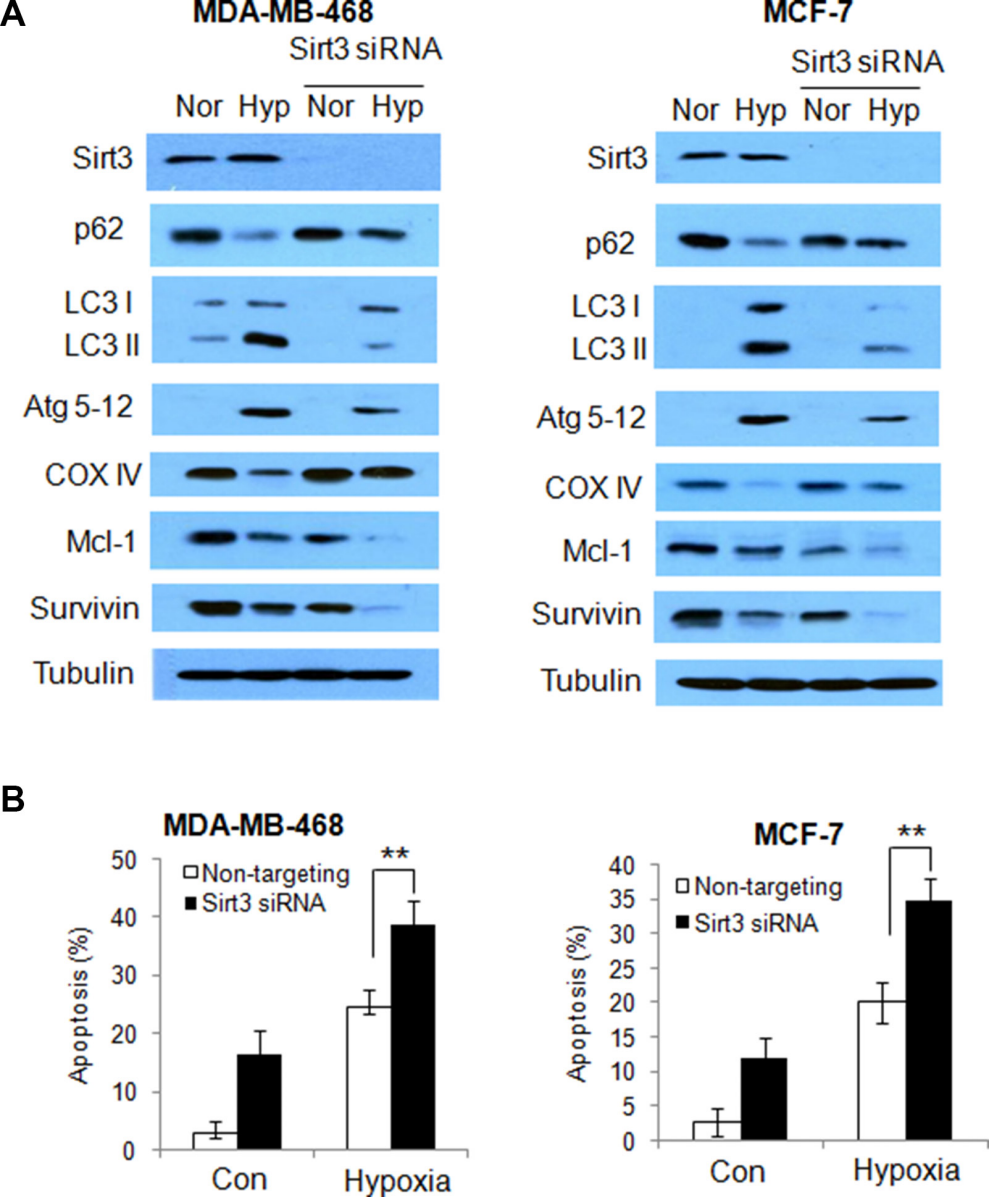
Silencing of Sirt3 decreases autophagy, but increases apoptosis in human breast cancer cells subjected to hypoxia (**A**) MDA-MB-468 or MCF-7 cells were transfected with a non-targeting RNA or a siRNA targeting Sirt3, followed by hypoxia treatment. The protein expressions of Sirt3, p62, LC3, Atg5-12, COX IV, survivin and Mcl-1 were examined by Western blot. Tubulin was used as a loading control. (**B**) Apoptosis was determined by flow cytometric analyses of Annexin staining.

To validate the role of Sirt3 in regulation of autophagy and apoptosis under stressful condition, we silenced the Sirt3 expression in the human breast cancer cells, following by hypoxia treatment, and the measured autophagic and apoptotic activities. As shown in Figure 7A, silencing of Sirt3 expression decreased autophagy in human breast cancer cells exposed to hypoxia, as indicated by an increase in p62 protein level, and decreases in LC3 and Atg5–12 proteins. Similarly, suppression of Sirt3 expression blocked the degradation of COX IV induced by hypoxia in human breast cancer cells (Figure 7A). Consistent with the results obtained with glioma cells, silencing of Sirt3 expression facilitated the down-regulation of Mcl-1 and survivin protein, and promoted the induction of apoptosis in human breast cancer cells under hypoxia (Figure 7B). These experiments demonstrated a critical role of Sirt3 in regulating autophagy and apoptosis in cancer cells.

## DISCUSSION

Autophagy is a biological mechanism for intracellular generation of nutrient molecules for cellular survival, and also plays an important role in removing damaged or excess organelles [16]. Mitophagy. often triggered by mitochondrial damage, is responsible for elimination of dysfunctional or impaired mitochondria [17]. In this study, we identified Sirt3 as a critical acitvator of mitophagy. Activation of mitophagy by Sirt3 appears to be mediated through increased interaction of VDAC1 with Parkin. Moreover, suppression of the Sirt3-mediated mitophagy promotes apoptotic cell death in the hypoxic cancer cells likely by enhancing the degradation of anti-apoptotic proteins Mcl-1 and survivin through a ROS-dependent proteasomal pathway.

These results reported here provide clear evidence for a role of Sirt3 in the regulation of autophagy. Sirt1 has been demonstrated to be a positive regulator of autophagy in mammalian cells and tissues [18]. Recently, Liang Q *et al*. reported that Sirt3-knockout mouse embryonic fibroblast cells exhibited increased autophagy compared with wild-type cells [19]. In contrast, we found that Sirt3 is a positive regulator of autophagy in cancer cells under hypoxia, and inhibition of Sirt3 blunts the induction of mitophagy triggered by hypoxia. VDAC1, an outer mitochondrial membrane protein, serve as the mitochondrial binding site for hexokinaseII [20]. The binding of hexokinaseII to the mitochondria mediated by VDAC contributes to cancer cell metabolism, and also inhibits damage to the mitochondria. Recent studies indicate that VDAC plays an important role in the induction of mitophagy by recruiting Parkin to defective mitochondria [15, 21]. Parkin, an E3 ubiquitin ligase, is recruited to the damaged mitochondria and promotes their degradation [22]. Sirt3 has been shown to promote detachment of hexokinaseII from VDAC1 [6, 14], and deacetylation of cyclophilin D by Sirt3 is involved in the dissociation of hexokinaseII from the mitochondria [14]. Consistent with these observations, we found that silencing of Sirt3 expression can blunt the redistribution of hexokinase from the mitochondria to the cytosol in hypoxic cells, and decreased interaction of hexokinase II with VDAC1 is accompanied by the increased interaction of VDAC1 with parkin in hypoxic cells. Depletion of Sirt3 decreases the association of VDAC1 and Parkin. These results suggest that Sirt3 may mediate the induction of mitophagy by promoting the dissociation of hexokinaseII from VDAC1, thus resulting in the binding of VDAC1 with Parkin.

Most studies indicate that the mitophagic response to mitochondrial damage is a pro-survival mechanism. Consistent with the reported observation [6], we found that depletion of Sirt3 increases apoptotic activity in hypoxic cancer cells. We further demonstrated that the down-regulation of Mcl-1 and survivin by the Sirt3 knockdown may account for the promotion of apoptosis. Mcl-1, a member of Bcl-2 family, plays a critical pro-survival role in the development and maintenance of both normal and malignant tissues, and is capable of blocking apoptosis induced by various apoptotic stimuli [23, 24]. Survivin, a structurally unique IAP, has been implicated in protection from apoptosis to stress. Mcl-1 and survivin are subject to rapid turnover through ubiquitin-dependent protein degradation by the 26S proteasome. We show here that proteasomal protease inhibitor, MG132, blocks the down-regulation of Mcl-1 and survivin in the Sirt3 knockdown cells, suggesting a role for Sirt3 in stabilizing Mcl-1 and survivin proteins. ROS, a natural byproduct of normal metabolism of oxygen, has important roles in cell signaling including protein degradation. Hypoxia-generated ROS increases Na, K-ATPase degradation via the ubiquitin-conjugating system [25]. ROS was shown to trigger Mcl-1 degradation through the proteasomal pathway [26, 27]. Here, we show that ROS promotes the degradation of Mcl-1 and survivin in the hypoxic tumor cells. As alterations of mitophagy and apoptosis have been known to be associated with tumor development, progression, and therapy, our study on the role of Sirt3 in regulating these two cellular processes provides evidence for exploiting Sirt3 as a new target for prevention and treatment of cancer.

## MATERIALS AND METHODS

### Cell lines and culture

Human glioblastoma cell lines T98G and LN229, and breast cancer cell lines MCF-7 and MDA-MB-468 were purchased from American Type Culture Collection. The cells were cultured in DMEM medium supplemented with 10% fetal bovine serum, 100 units/mL penicillin, and 100 μg/mL streptomycin. Cells were maintained at 37^°^C in a humidified atmosphere containing 5% CO_2_/95% air.

### Reagents and antibodies

MTT, Cycloheximide (CHX), MG132 and Flag antibody were purchased from Sigma. Antibodies to Sirt3, COX IV, hexokinase II, VDAC1, Mcl-1, survivin and prohibitin were purchased from Cell Signaling Technologies. LC3 antibody was purchased from nanoTools. α-tubulin antibody was purchased from Santa Cruz. Atg12 antibody was purchased MBL. p62 antibody was purchased fromEngo Life Science. All cell culture media were purchased from Invitrogen. Western blot reagents were obtained from Pierce Biotechnology.

### Western blot analysis

Cells were lysed in M-PER mammalian protein extraction reagent (Thermo Scientific) supplemented with a protease inhibitor cocktail (Roche) at room temperature for 5 minutes, followed by centrifugation at 14,000 × g for 10 minutes. Protein concentration of cell lysates was measured using the Bio-Rad DC assay reagent (Bio-Rad). Proteins (20–40 μg) were resolved on SDS-PAGE and transferred to PVDF membrane (Bio-Rad). The PVDF membranes were then incubated with respective antibodies in 3% BSA/TBST at 4^°^C for overnight, followed by incubation with secondary antibody at room temperature for 1 h. The protein signals were detected by ECL method.

### siRNA, shRNA and plasmid transfection

siRNA targeting Sirt3 was purchased from Santa Cruz Biotechnology. Scrambled non-targeting siRNA was used as a control. Transfection of siRNA was performed according to the manufacturer's protocol. Briefly, cells in exponential phase of growth were plated in six-well tissue culture plates at 1 × 10^5^ cells per well, grown for 24 h, and then transfected with siRNA using Oligofectamine and OPTI- MEM I– reduced serum medium. The concentrations of siRNAs were chosen based on dose-response studies. Flag-Sirt3 plasmid was a gift from Dr. Yue Xiong (University of North Carolina at Chapel Hill, Chapel Hill, NC, USA). Flag-Parkin plasmid is a gift from Dr. Lars Dreier (David Geffen School of Medicine at UCLA). Transfection of the plasmid was carried out using FuGENE6 transfection reagent (Promega) according to the manufacturer's protocol.

### Measurement of mitochondrial mass

Mitochondrial mass was measured by flow cytometry following NAO staining. Briefly, treated cells were collected and incubated with 10 nM NAO for 20 min at 37°C in a humidified atmosphere containing 5% CO_2_/95% air. At the end of incubation, the samples were analyzed on a Guava EasyCyte^TM^ Plus Flow Cytometry System (Millipore).

### Measurement of mitochondrial membrane potential

Mitochondrial membrane potential was measured by flow cytometry following JC-1 staining. Briefly, 4 μl of Guava cell staining solution (including JC-1 and MitoPotential7-AAD; Millipore) was added to 2 × 10^5^ cells (in 200 μl), and the cells were incubated with the reagent for 30 min at 37^°^C in a humidified atmosphere containing 5% CO_2_/95% air. At the end of incubation, the cells were analyzed by a Guava EasyCyte^TM^ Plus Flow Cytometry System (Millipore).

### ROS detection

ROS generation was measured using flow cytometry. Briefly, treated cells were collected and incubated with 10 μM DCF-DA (Sigma) for 30 minat 37^°^C in a humidified atmosphere containing 5% CO_2_/95% air. At the end of incubation, the samples were analyzed on an EasyCyte Plus Flow Cytometry System.

### Immunoprecipitation

Cells were lysed in the IP lysis buffer (150 mMNaCl, 50 mM Tris–HCl (pH 7.4), 1% Triton X-100) in the presence of protease inhibitors (Roche) on ice for 5 min, followed by centrifugation at 14 000 × g for 10 min at 4^°^C. Concentrations of proteins of the supernatants were determined, and samples containing equal amounts of proteins were preclearedusing protein A/G plus-agarose beads (Santa Cruz Biotechnology) prior to immunoprecipitation. The antibodies were then added to the precleared lysatesand incubated at 4^°^C for 1–3 h. After adding the fresh protein A/G plus-agarose beads, the lysates were further incubated for overnight. Finally, the beads were washed three times with the lysis buffer, and the proteins were eluted using the sample loading buffer.

### Cellular viability assay

Cell viability was measured by MTT assay. Briefly, cells were plated in 96-well tissue culture plates, and incubated at 37^°^C in a humidified atmosphere containing 5% CO_2_/95% air. The formazan product, formed after 4 h incubation with MTT, was dissolved in DMSO (Acros Organics, Cat. No. AC61042-0010) and read at 570 nm on a Victor3 Multi Label plate reader (PerkinElmer).

### Apoptosis assays

Apoptosis was determined by flow cytometric analysis of Annexin V and 7-AAD staining. Briefly, 100 μl Guava Nexin reagent (Millipore) was added to 1 × 10^5^ cells (in 100 μl) and the cells were incubated with the reagent for 20 min at room temperature in the dark. At the end of incubation, the cells were analyzed by a Guava EasyCyte^TM^ Plus Flow Cytometry System (Millipore).

### Statistical analysis

The difference between the samples with or without silencing of Sirt3 was analyzed using a unpaired two-tailed Student's *t*-test. All experiments were performed at least three times.
