# Correction: Sirt3-mediated mitophagy protects tumor cells against apoptosis under hypoxia

**DOI:** 10.18632/oncotarget.25620

**Published:** 2018-06-05

**Authors:** Aimin Qiao, Kuansong Wang, Yunsheng Yuan, Yidi Guan, Xingcong Ren, Lanya Li, Xisha Chen, Feng Li, Alex F. Chen, Jianda Zhou, Jin-Ming Yang, Yan Cheng

**Affiliations:** ^1^ Center for Bioresources and Drug Discovery and School of Bioscience and Biopharmaceutics, Guangdong Pharmaceutical University, Guangzhou 510006, China; ^2^ Department of Pharmacology, School of Pharmaceutical Sciences, Central South University, Changsha 410008, China; ^3^ Department of Pharmacology, The Penn State Hershey Cancer Institute, The Pennsylvania State University College of Medicine and Milton S. Hershey Medical Center, Hershey, PA 17033, USA; ^4^ Department of Pathology, Xiangya Hospital, Central South University, Changsha 410008, China; ^5^ Department of Pathology, Basic Medical School, Central South University, Changsha 410008, China; ^6^ Department of Plastic Surgery, The Third Xiangya Hospital, Central South University, Changsha 410008, China; ^7^ Engineering Research Center of Cell and Therapeutic Antibody, Ministry of Education, School of Pharmacy, Shanghai Jiao Tong University, Shanghai 200240, China; ^8^ Center for Vascular and Translational Medicine, The College of Pharmacy, Central South University, Changsha 410013, China; ^9^ The Third Xiangya Hospital, Central South University, Changsha 410013, China

**This article has been corrected**: The correct figure 5A cell line T98G is given below:

**Figure 5:**
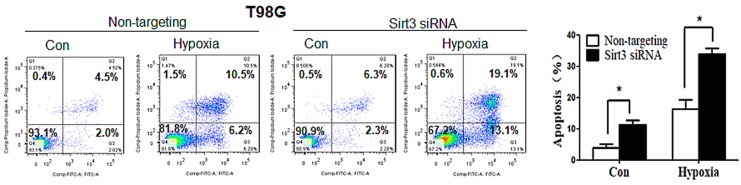
Suppression of Sirt3 augments the activation of apoptosis and increases the sensitivity of tumor cells to hypoxia (**A**) LN229 and T98G cells were transfected with a non-targeting RNA or a siRNA targeting Sirt3, followed by hypoxia treatment for 48h. Apoptosis was determined by flow cytometric analyses of Annexin staining.

The authors declare that these corrections do not change the results or conclusions of this paper.

Original article: Oncotarget. 2016; 7:43390-43400. https://doi.org/10.18632/oncotarget.9717

